# Supplementary material for: Thermodynamic Assessment of Triclocarban Dissolution Process in N-Methyl-2-pyrrolidone + Water Cosolvent Mixtures
Source: Molecules. 2023 Oct 22;28(20):7216. doi: 10.3390/molecules28207216 (PMC10609577; doi:10.3390/molecules28207216)
Supplement: Supplementary file 1 [file molecules-28-07216-s001.zip › molecules-2575826-supplementary.pdf]

Calibration curve of Triclcarban

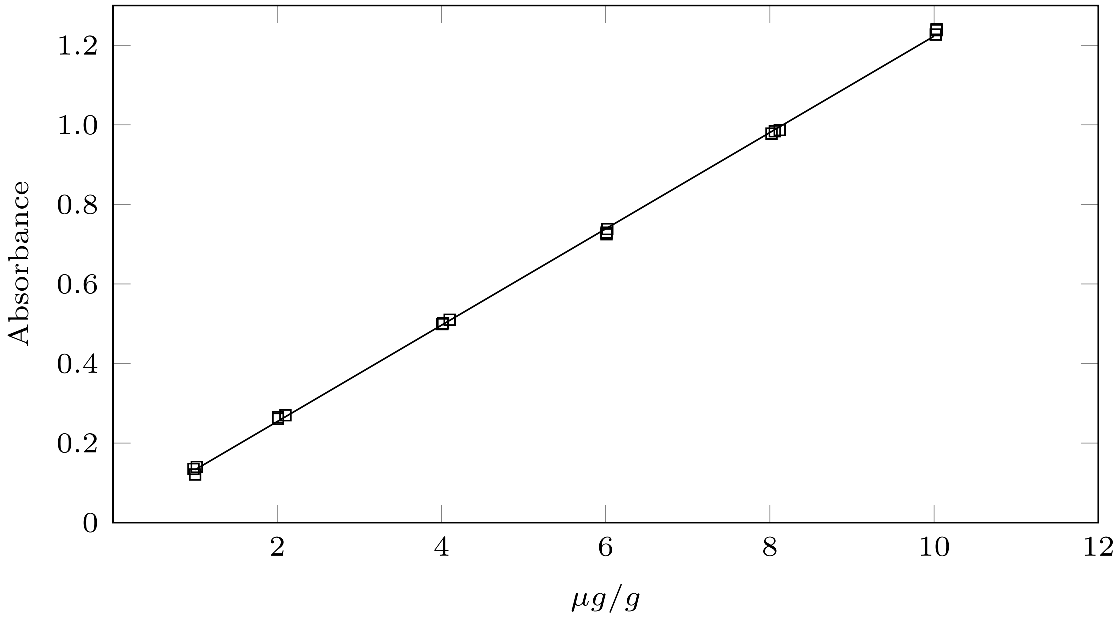

Figure S1: Calibration curve of triclocarban in absolute ethanol obtained at a wavelength of 265 nm.

Calibration curve equation

$$C = 8{,}255A - 0{,}0986 \tag{S1}$$

where C is concentration in $\mu g/g$ and A is an absorbance

.

Multiple R.	0.999793856
R squared.	0.999587755
Adjusted R square	0.999561989
Standard Error of the regression	0.008329394
Observations	18

Table S1: Regression statistics

	Degrees of freedom	Sum of Squares	Mean squared error	F	Significance F
Regression	1	2.691610217	2.691610217	38795.85348	1.63841E-28
Residual	16	0.001110061	6.93788E-05		
Total	17	2.692720278			
	Coefficients	Standard Error	t Stat	p-value	
Intercept	0.0117494	0.0037524	3.1311779	0.0064447	
slope	0.1211314	0.0006150	196.96663	1.63841E-28	

Table S2: ANOVA
